# Fabrication of Superhydrophobic Ultra-Fine Brass Wire by Laser Processing

**DOI:** 10.3390/ma18071420

**Published:** 2025-03-23

**Authors:** Jing Sun, Hao Huang, Jiajun Ji, Chen Zhang, Binghan Wu, Hao Liu, Jinlong Song

**Affiliations:** 1 State Key Laboratory of High-Performance Precision Manufacturing, Dalian University of Technology, Dalian 116024, China; sunjing@dlut.edu.cn (J.S.); 15641630588@163.com (H.H.); jiajunji1999@163.com (J.J.); 935381361@mail.dlut.edu.cn (C.Z.); 13280607167@163.com (B.W.); 15141623006@163.com (H.L.); 2 Key Laboratory for Micro/Nano Technology and System of Liaoning Province, Dalian University of Technology, Dalian 116024, China

**Keywords:** superhydrophobic, ultra-fine metal wire, laser processing, micro morphology

## Abstract

Superhydrophobic metal wires have shown great application prospects in oil–water separation, anti-corrosion, anti-icing, and other fields due to their excellent water repellency. However, how to fabricate a superhydrophobic surface on ultra-fine metal wire remains a challenge. Here, we proposed a method using laser processing to efficiently fabricate superhydrophobic ultra-fine brass wire. Firstly, we analyzed the mechanism of the laser processing of curved surfaces and designed a controllable angle rotation fixture to avoid the machining error caused by secondary positioning in the machining process. Then, we investigated the influences of the laser power, scanning speed, and scanning times on the surface morphology and wettability of the ultra-fine brass wire. The optimal laser processing parameters were obtained: laser power of 6 W, scanning speed of 500 mm/s, and scanning time of 1. After low surface energy modification, the water contact angle and surface roughness *S*a of the ultra-fine brass wire were 156° and 1.107 μm, respectively. This work is expected to enrich the theory and technology for fabricating superhydrophobic ultra-fine brass wire.

## 1. Introduction

The superhydrophobic metal surface, inspired by the lotus leaf, has captivated researchers due to its excellent water repellency and has shown great application prospects in fluid transportation [1,2,3], anti-icing [4,5,6], and other fields [7,8,9,10]. Wang et al. used the nanosecond laser-chemical treatment method to fabricate three different periodic surface patterns on the surface of 1095 carbon steel sheets. The maximum water contact angle was as high as 159°, and the corrosion resistance was significantly improved [11]. Wang et al. also fabricated a superhydrophobic surface on SS304L by an innovative ultrasonic-assisted laser-silicone oil (ULSO) treatment method, which greatly improved its corrosion resistance [12]. Ganesh et al. used the nanosecond laser to form a superhydrophobic texture on the surface of 316 stainless steel. After 30 days of natural storage, the water contact angle can reach 157° [13]. With the development of technology, micromanufacture is the mainstream direction of the modern manufacturing industry. The superhydrophobic ultra-fine metal wires have advantages in the processing scale compared with the superhydrophobic surface, and can achieve more precise work [14,15]. In addition, superhydrophobic metal mesh woven by superhydrophobic metal wires can be used to efficient oil–water separation and fog collection due to its structure with numerous pores [16,17,18,19]. Therefore, how to fabricate the micro/nano-structure, which is necessary for superhydrophobicity, on metal wire has attracted attention.

At present, most researchers have fabricated micro/nano-structures on the surface of metal wire via chemical etching [20,21], the hydrothermal method [22,23], electrochemical deposition [24,25], and other methods [26] to obtain superhydrophobic metal wire. However, in the above methods it was difficult to uniformly construct the necessary micro/nano-structure for superhydrophobic surfaces on the single ultra-fine metal wire. A single ultra-fine metal wire plays a decisive role in the machining process as a tool or cathode, especially in some precision special processing fields [27,28,29]. Due to the hydrophilicity of ultra-fine metal wires, they could have a significant impact on machining accuracy. Researchers have made significant efforts to address the aforementioned issues, until 2022 when Liu et al. [30] fabricated hydrophobic ultra-fine metal wire by electrochemical machining to improve its machining accuracy as a cutting tool. Therefore, how to efficiently fabricate superhydrophobic ultra-fine metal wire remains a challenge.

In this study, we proposed a laser processing method to fabricate superhydrophobic ultra-fine metal wire. We first analyzed the mechanism of the laser processing of curved surfaces and designed a special laser processing fixture for ultra-fine brass wire. The single factor experiments were conducted to explore the optimal laser processing parameters: laser power, scanning speed, and scanning time. The influence of laser parameters on the surface microstructure and wettability of the ultra-fine brass wire during processing was further studied by characterization analysis. This technique provides a new method for the efficient preparation of superhydrophobic ultra-fine metal wire.

## 2. Experimental Details

### 2.1. Materials

Commercially available brass wires (200 μm diameter, 70% Cu, 30% Zn) and brass plates (20 mm × 20 mm × 2 mm, 70% Cu, 30% Zn) were purchased from Kunshan Shunxin Mechanical and Electrical Equipment Co., Ltd. (Shenzhen, China). Acrylic board was purchased from China Shanxi Circumference New Material Co., Ltd. (Xi’an, China). Sandpaper (1200 # and 2000 #) was purchased from Shanghai Xinyi Industrial Co., Ltd. (Shanghai, China). Fluoroalkylsilane (FAS, C_8_F_13_H_4_Si(OCH_2_CH_3_)_3_) was purchased from Degussa (Frankfurt, Germany). The absolute alcohol and hydrochloric acid (HCl) used in the experiment were purchased from Bono Chemical Reagents Co., Ltd. (Dalian, China).

### 2.2. Fabrication of Superhydrophobic Brass Wire

**Figure 1:**
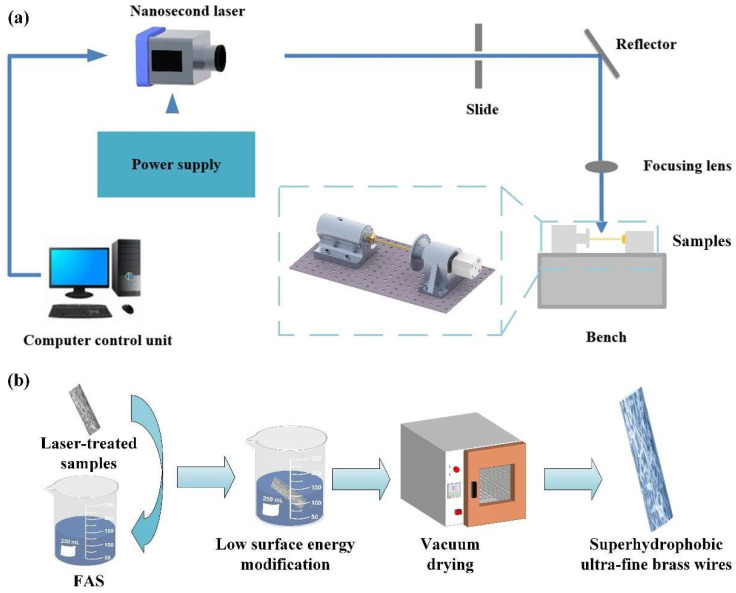
Superhydrophobic ultra-fine brass wire preparation process diagram: (**a**) laser processing, and (**b**) subsequent low surface energy modification.

Since the brass has excellent thermal conductivity, ductility, wide adaptability, and a low price compared with other metal materials, we chose ultra-fine brass wire for fabrication. The brass wire was pretreated before the laser processing test. The brass wire was cut off by scissors and polished twice separately with 1200 # and 2000 # sandpaper [31]. The polished copper wire was cleaned twice in an ultrasonic cleaner (LT-05C, Longbiao Electric Co., Ltd., Jinan, China). For the first time, deionized water was used to clean inorganic impurities such as debris particles, and for the second time, absolute alcohol was used to clean potential organic impurities such as oil stains. The cleaning time was 20 min each. The cleaned brass wire was dried by an electrothermal constant temperature oven (DHG-9023A, Jinghong experimental ablation process, computer-controlled). After drying, the brass wire was clamped with a special fixture. The nanosecond laser marking system (wavelength 1064 nm, repetition rate 20 kHz, pulse duration 100 ns, spot diameter about 50 μm, optical focusing focal length, and scanner head 19.5 mm, maximum average power 30 W, SK-CK30, Sanke Laser Technology Co., Ltd., Shanghai, China) was used to process the sample. The laser scanning pattern was set to a square with a size of 60 mm × 60 mm by a computer drawing software. The scanning line spacing was set to 50 μm. The pattern was filled horizontally and vertically to ensure that the sample surface could fully absorb the laser energy. Then, the micro/nano-structure was fabricated on the sample surface [32]. The processes of fabricating superhydrophobic ultra-fine brass wire are shown in Figure 1a. The subsequent process was shown in Figure 1b. The ultra-fine brass wire processed by the nanosecond laser, was cleaned by ultrasonic cleaner for 10 min, and immersed in FAS solution with a concentration of 1 wt % for 1 h [33]. Finally, the superhydrophobic ultra-fine brass wire samples were obtained by vacuum drying in an oven at 80 °C for 20 min.

### 2.3. Characterization

The surface microstructure of the samples was observed using a scanning electron microscope (SEM, SUPRA 55 SAPPHIRE, Oberkochen, Germany). The 3D surface profiler (Zygo, NewView9000, Middletown, CT, USA) was used to observe the surface roughness of the laser processing area of the brass plate. For the samples fabricated under the same parameters, at least four different points were selected for measurement, and the average value was finally taken to reduce the measurement error. The surface roughness of the brass wire sample was measured using a laser confocal microscope (CLSM, LSM900, Oberkochen, Germany). The chemical composition of the superhydrophobic surface was measured using an energy dispersive spectrometer (EDS, SUPRA 55 SAPPHIRE, Oberkochen, Germany). The crystal structure of the superhydrophobic surface was measured using an X-ray diffractometer (XRD, Empyrean, Alemlo, Netherlands). The optical contact angle meter (Solon, SL200KS, Boston, MA USA) was used to drop 5 μL of water droplets on the surface of the prepared sample at room temperature, and the contact angle (CA) of the sample was measured. Samples under the same laser parameters were measured at least 5 times, and the average value was taken for data processing. A camera (D7200, Nikon, Tokyo, Japan) was used to record the experimental process.

## 3. Results and Discussion

### 3.1. Processing Method and Fixture Design

**Figure 2:**
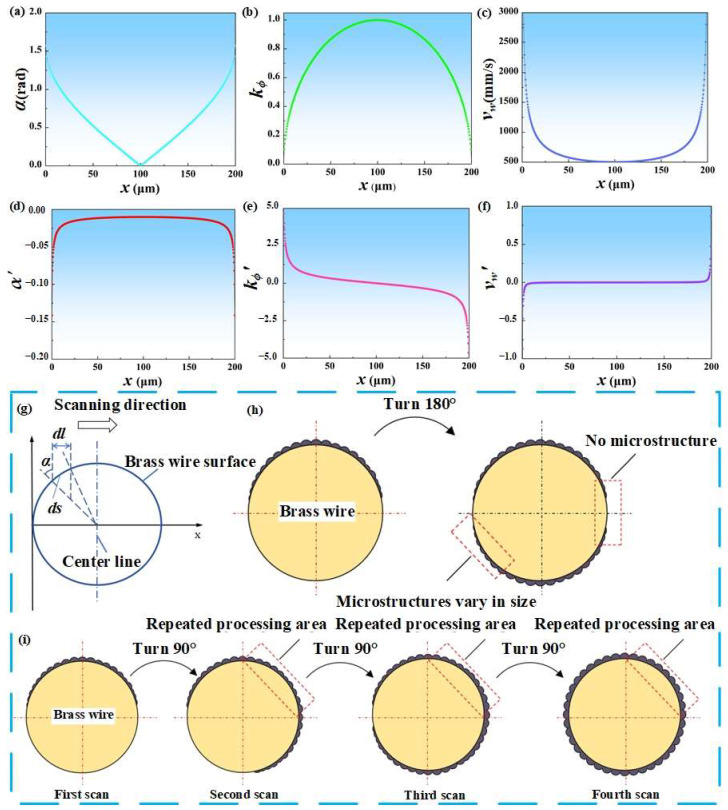
Mechanism analysis of laser processing brass wire: (**a**) The image of *α* changing with *x*. (**b**) The image of *k*_*ϕ*_ changing with *x*. (**c**) The image of *v_ω_* changing with *x*. (**d**) The image of *α*′ changing with *x*. (**e**) The image of *k*_*ϕ*_′ changing with *x*,. (**f**) The image of *v*_ω_′ changing with *x*. (**g**) The schematic diagram of the relative position of the incident angle *α* and the spot. (**h**) Initial processing scheme. (**i**) The final processing scheme.

Due to the small diameter of brass wire, higher laser power and scanning times during laser processing can easily cause the brass wire to melt. At the same time, the brass wire should be kept taut during laser processing to ensure that the surface can uniformly absorb the energy of the laser. As the processing progresses, the mechanical properties such as the toughness of the copper wire become worse, and it is also possible to break or fail to straighten. Therefore, exploring an appropriate processing method and designing a suitable fixture have become the core issues of the test. The schematic diagram of the laser processing brass wire surface is shown in Figure 2g. According to the current literature, the absorption rate of the material to the laser is mainly determined by the beam wavelength, incident angle and polarization characteristics [34,35,36,37,38,39]. Since the laser wavelength used in the experiment and the polarization characteristics of the material have been determined, the incident angle is explored. The relationship between the incident angle *α* and the position *x* of the laser spot is shown in Equation (1).

(1) α=|arcsin(1−2x/D)|

where *D* represents a diameter of brass wire is 200 μm. The relationship between the incident angle *α* and *x* is shown in Figure 2a. The laser incidence angle and incidence angle change rate (curve slope) increase from the center of the brass wire to both ends. It can be seen that the absorption rate of laser energy on the surface of brass wire shows a decreasing trend from the center to the edge. When *x* is 0 or 200 μm, the incident angle reaches almost 90°, and the material hardly absorbs laser energy. It shows that if the surface of brass wire is fabricated by plane processing method, the edge of brass wire is difficult to be fabricated and microstructure cannot be formed.

The key to fabricating the microstructure on the surface of brass wire is the actual laser energy density on the surface. In order to facilitate the subsequent calculation, a small square area with a side length of d*l* is arbitrarily selected in the laser spot, and its area is d*l* × d*l*. Then, the projection of the area on the surface of the brass wire should be an arc surface, and the arc surface area is d*s* × d*l*, where the calculation formula of d*s* is shown in Equation (2).

(2) $$ds=\frac{\pi D}{360}d\alpha =\frac{\pi dx}{180\sqrt{1-{\left(1-2x/D\right)}^{2}}}$$

supposing the total laser energy is *E*. In the square region, the calculation formula of the initial laser energy density *ϕ*_1_ (the laser energy density at *α* = 0°, that is, the energy density when processing the plane sample) is shown in Equation (3).

(3) $$\phi_{1}=\frac{E}{dl\times dl}$$

combining Equations (2) and (3), the actual laser energy density *ϕ*_2_ on the arc surface is shown in Equation (4).

(4) $$\phi_{2}=\frac{E}{ds\times dl}=\frac{180\phi 1dl\sqrt{1-{\left(1-2x/200\right)}^{2}}}{\pi dx}$$

The ratio of the initial laser energy density to the actual laser energy density is defined as the laser energy density attenuation rate *k*_*ϕ*_, and its calculation formula is shown in Equation (5).

(5) $$k\phi =\frac{180\sqrt{1-{\left(1-2x/200\right)}^{2}}}{\pi }$$

It can be seen from Figure 2b that the farther away from the geometric center of the brass wire, the larger the attenuation rate of the laser energy density. At the same time, the larger the slope of the curve represents the faster the attenuation, that is, the lower the absorption rate of the laser energy by the surface material at the edge of the brass wire. This is the same as the above trend of exploring the influence of incident angle changes on surface material processing.

In addition to the above two points, because the processing surface is a curved surface, the actual processing path of the laser spot is an arc, and its scanning speed is different from that on the plane. The actual scanning speed is represented by the curve speed *v*_ω_, and its calculation formula is shown in Equation (6), and its relationship with the spot position *x* is shown in Figure 2c.

(6) $$v_{\omega }=\frac{ds}{dt}=\frac{\pi Dv}{360}\frac{1}{\sqrt{Dx-x^{2}}}$$

According to Figure 2c, it can be seen that *v*_ω_ shows an increasing trend from the center to the edge of the brass wire. The larger the curve speed, the shorter the time for the surface material of the brass wire to absorb laser energy, and the worse the processing effect. Based on the above three points, the key to the overall process flow is the uniform processing at the edge of the brass wire, and a uniform microstructure is also formed at the edge of the brass wire. If the processing method shown in Figure 2h is adopted, one side is turned 180° and then processed again; then, the microstructure will not be formed at the edge of the brass wire, and the hydrophobicity of the subsequent brass wire will also be affected, and superhydrophobicity cannot be achieved. In order to solve this problem, continue to explore the appropriate processing methods: the process is as follows.

By deriving Equations (1), (5) and (6), the rate of change in the laser incident angle (*α*′), the rate of change in the laser energy density attenuation rate (*k*_*ϕ*_′), and the rate of change in the laser actual scanning speed (*v*_ω_′) on the surface of the wire electrode can be obtained as shown in Equation (7), Equation (8), and Equation (9), respectively.

(7) $$\alpha^{\prime }=-\frac{1}{\sqrt{Dx-x^{2}}},0\leq x\leq D$$

(8) $$k_{\phi }^{\prime }=\frac{180}{\pi }\frac{D-2x}{\sqrt{Dx-x^{2}}}$$

(9) $$v_{\omega }^{\prime }=-\frac{\pi Dv}{720}\frac{D-2x}{{\left(Dx-x^{2}\right)}^{\frac{3}{2}}}$$

The relationship between them and the spot position *x* is shown in Figure 2d–f, respectively. Combined with Figure 2d–f, The enlarged image of Figure 2f can be seen in Figure S1 in the Supplementary Materials. It can be seen that when *x*∈[25, 175], the values of *α*′, *k*_*ϕ*_′ and *v*_ω_′ are relatively small, and the change range is not large, and the image is relatively stable. The absorption effect of brass wire on laser energy is basically the same in this position range, and relatively uniform microstructure can be obtained. The range of the above *x* is more than 75% of the diameter of the brass wire. When laser scanning the surface of brass wire, *α*′, *k*_*ϕ*_′, and *v*_ω_′ do not change much in the part of the scanning area exceeding 75%, that is, the arc surface corresponding to the central angle of 135°. Therefore, the processing method shown in Figure 2i is proposed, and the processing is performed once every 90°, a total of four times. The processing was carried out according to this method, and the surface roughness was measured after each laser scanning. The surface roughness values after the four scans were shown in Table S1 of the Supplementary Materials (Positions A, B, C and D correspond to the first to fourth scans in Figure 2i, respectively). It should be noted that there will be repeated processing areas in this method. When the laser energy density exceeds the strong ablation threshold of the material, the repeated processing area may have a great influence on the shape and size of the microstructure. Therefore, the method shown may need to be carried out at low laser power, which provides a guiding role for subsequent parameter exploration.

In the fixture design, there are three core requirements. The first is to keep the brass wire straight in the whole processing project to ensure that the distance between the laser and the material surface is always equal to the laser focal length, so as to achieve uniform processing. The second is to flip the brass wire without repeated positioning. This is because if repeated positioning is required, there will inevitably be some wear and processing deviations during the disassembly process. The position of the brass wire relative to the fixture will inevitably change, resulting in uneven distribution of the processed surface micro/nano-structure, which means that the processed brass wire cannot meet the requirements. The third is that the fixture can realize 90° fixed angle rotation. Combined with the above requirements, the main body of the designed fixture is divided into two parts: the indexing wheel and the connecting base, which can realize the fixed clamping of the brass wire and keep the brass wire straight during the processing. There is a card slot in the dividing wheel every 30°. After the brass wire is clamped and fixed, the angle can be adjusted to 30° as the minimum unit without secondary disassembly, which meets the above core requirements for fixture design.

### 3.2. Laser Power

In this paper, the effects of laser power, scanning speed, and scanning times on the formation of the microstructure and wettability of ultra-fine brass wire surface were investigated. A nanosecond laser has the characteristics of Gaussian energy distribution, and its laser center intensity is greater than the surrounding intensity [40,41]. When it acts on the surface of the material, the absorption rate of the material surface to the laser is also different. The results show that the laser energy is absorbed by the surface of the material without exceeding the ablation threshold, and the surface temperature of the ultra-fine brass wire increases continuously. With the increase in energy, after reaching a certain ablation critical point, the surface layer of ultra-fine brass wire begins to ablate. When the energy density of the laser is higher, more energy will enter the range of laser ablation, resulting in an increase in the range of laser ablation and a deeper depth. Due to the difference between the internal and external light intensity of the light spot, the temperature difference in each part after melting occurs, which in turn affects the surface tension of each part, so that the fluid flows from the high temperature zone to the low temperature zone [42]. After the surface is cooled, the fluid will be transformed into the microstructure. That is to say, as the laser energy increases, the depth of laser ablation becomes deeper and the microstructure becomes rougher.

**Figure 3:**
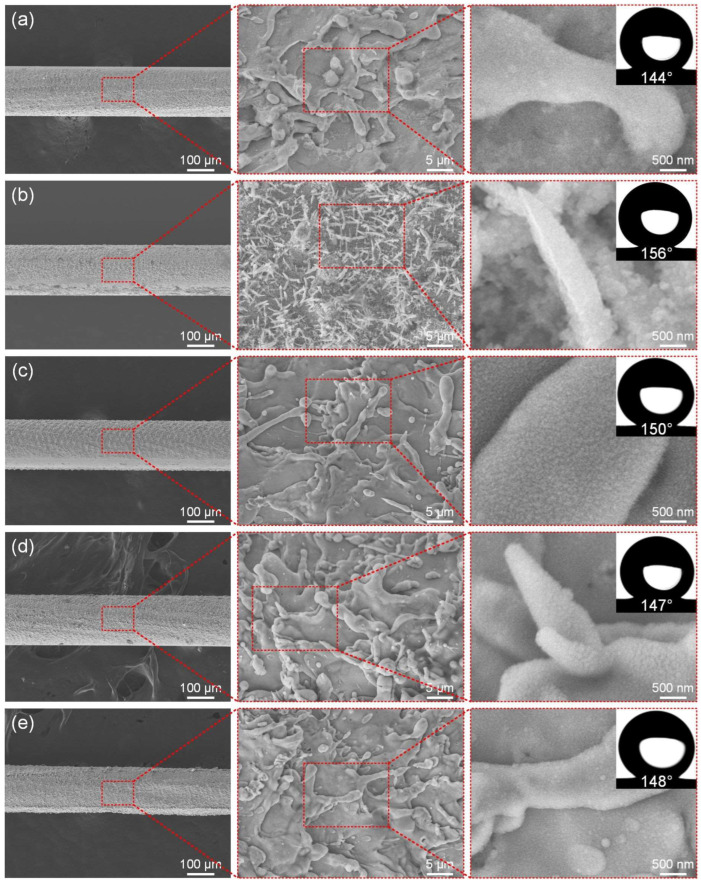
The SEM of superhydrophobic ultra-fine brass wire fabricated by different laser powers: (**a**) 3 W, (**b**) 6 W, (**c**) 9 W, (**d**) 12 W, and (**e**) 15 W.

The laser power directly determines the energy density of the laser, which in turn affects the surface microstructure of the fabricated ultra-fine brass wire and ultimately affects its wettability. The initial scanning speed is set to 500 mm/s, the fixed scanning line spacing is 50 μm, and the scanning time is 1. Figure 3a–e show the microstructure of the ultra-fine brass wire surface at different laser powers (3–15 W) and the best hydrophobic surface formed after FAS modification. According to Figure 3a, the density of the surface microstructure of the ultra-fine brass wire manufactured at low power (3 W) was not high. At the same time, due to the lack of good micro/nano-structures, its hydrophobicity was not good, with a water contact angle of 144°, which falls short of the criteria for superhydrophobicity. After appropriately increasing the laser power to 6 W, according to Figure 3b, the spike structure was obtained on the ultra-fine brass wire surface. The reason for this structure may be as follows: when the laser energy density acting on the surface of the sample exceeds the weak ablation threshold (melting threshold) of the material, a metal melting layer of tens to hundreds of nanometers thick will be formed in the processing area. Because the energy density of the center of the laser spot is much higher than that of the edge, the thickness of the metal melting layer in the processing area of the center of the laser spot is larger, and a temperature gradient decreasing from the center to the edge will be formed [43,44]. With the movement of the laser spot, the metal melt will solidify again. Under the combined action of temperature gradient and melt surface tension, a spike microstructure was formed. According to the Cassie–Baxter model, its theoretical superhydrophobic performance should be greatly improved, and the experimental measurement is also consistent with the theory [45,46,47]. The contact angle can reach 156°, which is the optimal parameter of the test group. When the laser power continues to increase to 9 W, according to Figure 3c, the spike structure on the surface of the ultra-fine brass wire disappeared, and the superhydrophobic performance also decreased slightly, and the contact angle decreased to 150°, but it could still maintain superhydrophobicity. According to Figure 3d, when the laser power continued to increase to 12 W, the surface of the brass wire exhibited a mastoid microstructure. This was due to the increase in laser power and the increase in laser energy. The original spike structure was recast by fusing to form a mastoid structure. The number of microstructures increased compared with that of 9 W, and the microstructure size also increased slightly, but the superhydrophobic performance decreased slightly. The contact angle was 147°. Continuing to increase the laser power, as shown in Figure 3e, there was little change in the microstructure morphology of the brass wire surface, which remained a mastoid structure. However, due to the high laser power, the surface of the brass wire had obvious processing dent defects, and the tensile strength was greatly reduced. It is difficult to put into subsequent use, so the processing of the brass wire should adopt a low-power laser parameter. Combined with the above content, as the laser power increased, the number of microstructures on the surface of the brass wire sample showed an increasing trend, and its appearance transitioned from a spike structure to a mastoid structure. When the laser power was 6 W, the surface of the brass wire exhibited a spike structure. In terms of wettability, as the power increased, the contact angle first increased rapidly and then tended to be gentle and slightly decreased. The water contact angle of the sample fabricated by the laser power of 6 W was 156°. We continued to increase the laser power, although the sample also had superhydrophobicity and the contact angle was not much different. Therefore, from the perspective of energy conservation, the laser power of 6 W was selected as the optimal parameter for subsequent experiments.

### 3.3. Laser Scanning Speed

Another very important parameter of nanosecond laser processing is the laser scanning speed. Since the nanosecond laser used is a pulse type laser, the scanning speed determines the length of the laser residence time at each position. When the laser processing energy is greater than the ablation energy threshold, the micro/nano-structure will be continuously generated by the laser ablation. Reducing the scanning speed means that the laser energy has a longer action time when processing each position. In order to explore the influence of the nanosecond laser scanning speed on the surface of the brass wire samples, the above optimal laser power of 6 W, fixed scanning line spacing of 50 μm, and scanning time of 1 were used to explore the influence of scanning speed on the surface microstructure and wettability of ultra-fine brass wire.

**Figure 4:**
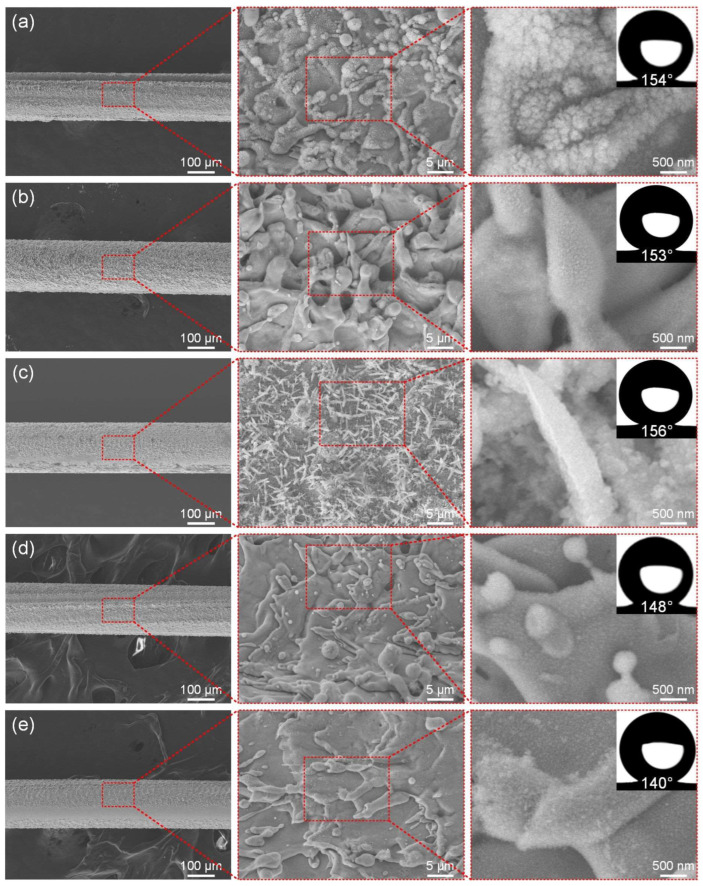
The SEM of the superhydrophobic ultra-fine brass wire fabricated by different scanning speeds: (**a**) 300 mm/s, (**b**) 400 mm/s, (**c**) 500 mm/s, (**d**) 600 mm/s, and (**e**) 700 mm/s.

Similarly to exploring the influence of power, Figure 4a–e show the microstructure of the ultra-fine brass wire surface at different scanning speeds (300–700 mm/s) and the best hydrophobic surface formed after FAS modification. When the scanning speed was low, it was obvious that the laser stayed on the surface of the ultra-fine brass wire for a longer time, the material absorbed the laser energy more fully, and the surface formed a denser microstructure. As shown in Figure 4a, at the low scanning speed (300 mm/s), the surface of the brass wire formed the mastoid microstructure, and there were smaller micro/nano particle structures on the mastoid, which is a typical micro/nano-structure [48]. In theory, good superhydrophobic properties should be obtained after FAS modification. The fact also conforms to the theory. The water contact angle of the structure can reach 154°, but the firmness and durability of the particles were not good. This situation could be attributed to the excessive laser energy absorption by the surface of the ultra-fine brass wire, which reduced its toughness as a result of prolonged laser processing, and caused its surface to exhibit obvious processing dents. According to Figure 4b, as the scanning speed gradually increased, the particle structures gradually disappeared, and the mastoid structure also changed to the spike structure, and the superhydrophobic performance of the ultra-fine brass wire surface was improved. According to Figure 4d, at the scanning speed of 600 mm/s, the surface microstructure of the ultra-fine brass wire became significantly less pronounced, with spike microstructures nearly vanishing and only faint contours remaining visible. The lack of micro/nano-structures will inevitably lead to a decrease in superhydrophobic properties. The water contact angle of the sample under this parameter was reduced to 148°, which had lost superhydrophobicity. Further increasing the scanning speed, according to Figure 4e, the machining quality of the ultra-fine brass wire surface became extremely uneven, and some surfaces did not even have a microstructure after processing, and the water contact angle of the sample was further reduced to 140°. In summary, as the scanning speed increased from 300 mm/s to 500 mm/s, the micro/nano-structure formed on the surface of the ultra-fine brass wire changed from the mastoid to the spike. As the scanning speed continued to increase to 700 mm/s, the surface micro/nano-structure gradually disappeared. The surface contact angle increased slowly and then decreased rapidly. Under the parameter of 500 mm/s, the maximum contact angle was 156°, and the superhydrophobic performance was the best. Therefore, the scanning speed of 500 mm/s was selected as the better laser parameter in the subsequent tests.

### 3.4. Laser Scanning Times

**Figure 5:**
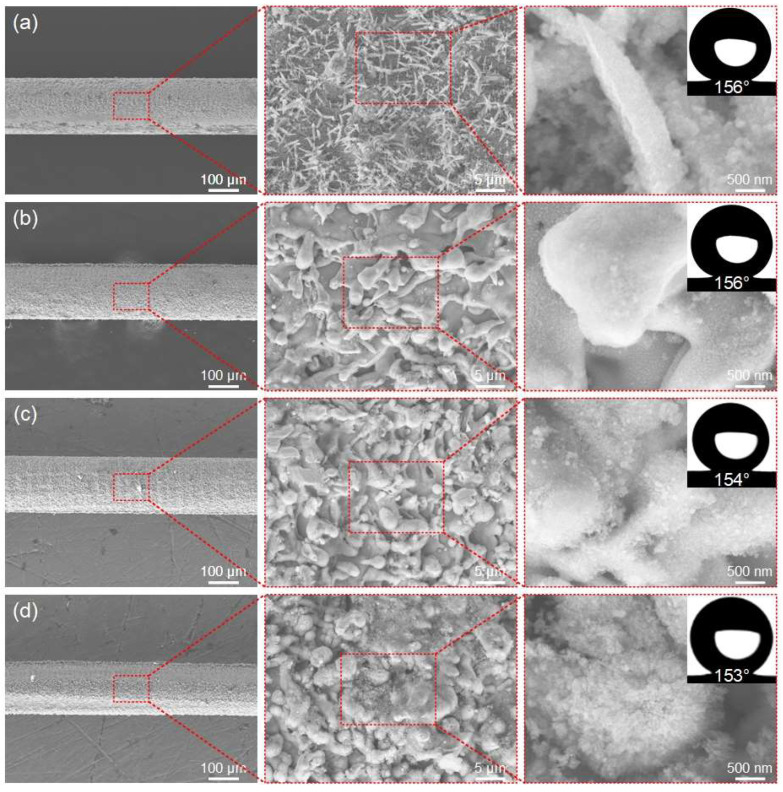
The SEM of the superhydrophobic ultra-fine brass wire fabricated by different scanning times: (**a**) 1 time, (**b**) 2 times, (**c**) 3 times, and (**d**) 4 times.

Finally, the effect of the scanning times on the micro/nano-structure of the ultra-fine brass wire surface and the hydrophobicity after FAS modification were investigated. The scanning times here refers to the number of processing times for the same area, rather than the number of flips mentioned in the previous section. The laser power was selected as 6 W, the scanning speed was 500 mm/s, and the scanning line spacing was fixed as 50 μm. The micro/nano-structure of the ultra-fine brass wire surface under different scanning times (1 to 4 times) and the best hydrophobic surface formed after FAS modification were shown in Figure 5a–d.

The surface of the ultra-fine brass wire produced through a single nanosecond laser scan resulted in the spike structure mentioned earlier. According to Figure 5b, when the scanning times was 2, there was no obvious processing defect in the macroscopic structure of the ultra-fine brass wire. In the microstructure, the original spike microstructure on the surface of ultra-fine brass wire became a mastoid microstructure. The specific element composition analysis of these two microstructures will be described in the next section. It was similar to the mastoid microstructure mentioned in the previous two sections. The structure was very dense, and the aforementioned micro/nano particle structures also adhered to its surface. These complex and dense microstructures have good superhydrophobicity after FAS low surface energy modification. After repeated measurements, the average water contact angle could reach 156°. However, considering the energy consumption and processing time, the scanning time of 1 could meet the superhydrophobic requirements. According to Figure 5c, when the laser scanning times was increased to three times, the macroscopic structure of the sample had some processing defects. In terms of the microstructure, it was still a composite micro/nano-structure with an interlaced spherical microstructure and spike microstructure. However, the proportion of spherical microstructure increased, and the proportion of spike microstructure decreased. The wettability was not much different from that of the sample obtained by laser scanning twice. The water contact angle after FAS modification could reach 154°, and it also had good superhydrophobicity. However, due to the macroscopic defects on the surface, it was difficult to put into practical application. According to Figure 5d, when the scanning times reached 4, noticeable processing defects could be observed on the surface of the ultra-fine brass wire at the macroscopic level. Some of the surface of the cylinder was eliminated by the nanosecond laser, and the ultra-fine brass wire was no longer a regular cylinder. On the microstructure, the surface of the ultra-fine brass wire had been unable to observe the traces of the spike microstructure. Instead, it was replaced by the density microsphere structure, which was also a good micro/nano-structure. The water contact angle could reach 153° after low surface energy modification. Although the ultra-fine brass wire fabricated under these parameters was capable of meeting the superhydrophobicity requirements, in order to improve processing efficiency, we decided that the optimal laser processing parameter of scanning times was 1.

### 3.5. Chemical Composition Analysis

**Figure 6:**
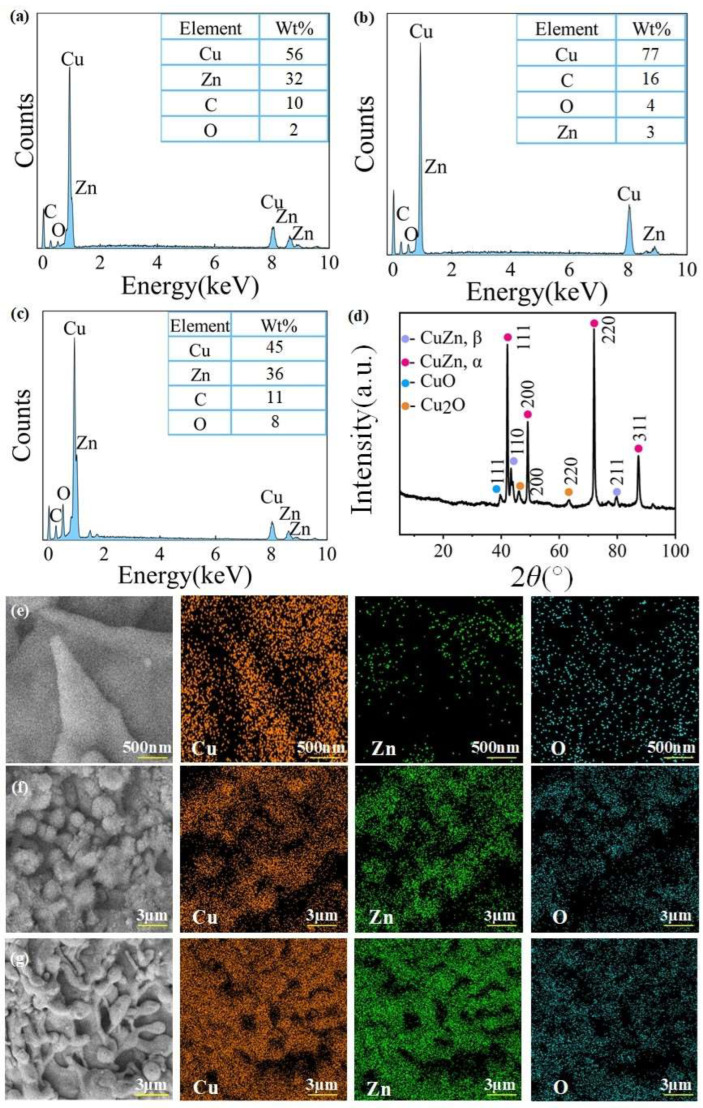
The chemical composition analysis of the microstructure on the surface of ultra-fine brass wire after laser processing: (**a**) EDS spectrum of the unprocessed ultra-fine brass wire, (**b**) EDS spectrum of laser power was 6 W, (**c**) EDS spectrum of laser power was 12 W, (**d**) XRD diffraction patterns, (**e**) EDS mapping of spike microstructure, (**f**) EDS mapping of mastoid microstructure, and (**g**) EDS mapping of spherical microstructure.

We used 250,000 points to accumulate EDS spectra. The signal accumulation statistic (the maximum count values) for EDS measurements was 18,623, the peak-to-background ratio for oxygen EDS signal was 3. The EDS spectrum of the surface chemical composition analysis of ultra-fine brass wires under different processing parameters was shown in Figure 6a–c. It can be seen that compared with the unprocessed ultra-fine brass wire, the content of Cu and C elements on the surface of the spike structure formed after 6 W laser power processing increased sharply. The content of Cu increased from 56% to 77%, the content of C increased from 10% to 16%, and the content of O increased from 2% to 4%. However, the content of Zn decreased from 32% to 3%, and the Zn was almost not detected in the spike structure, indicating that the main component of the spike structure may be Cu oxide. The subsequent EDS mapping analysis and XRD diffraction patterns also verified this conjecture. Upon the disappearance of the spike structure, the Cu content decreased significantly from 77% to 44%, while the Zn content rose from 3% to 35%, approaching its level on the unprocessed surface. Meanwhile, the O content increased from 3% to 9%, indicating that a significant amount of Cu and Zn oxides was formed. The possible reason for this phenomenon is that during laser processing, high-energy laser beams can cause local heating and melting of the brass surface. Zn has a lower melting point (419.5 °C) and boiling point (907 °C) compared with Cu (melting point: 1085 °C, boiling point: 2562 °C) [49,50]. As a result, Zn is more susceptible to ablation and evaporation under laser irradiation. The laser energy preferentially removes Zn from the surface due to its lower thermal stability. This selective ablation leads to a significant reduction in Zn content on the surface, while Cu, being more thermally stable, remains largely intact. When the laser power continues to increase, due to the small surface area of the ultra-fine brass wire, the surface temperature will continue to rise, and copper and its oxides will sublime, revealing the original surface of the copper wire again. Therefore, the Zn content will return to normal.

To study the specific components of the spike, spherical, and mastoid microstructures mentioned above, the three structures were subjected to EDS mapping analysis, and the results were shown in Figure 6e–g. It can be seen from Figure 6e that the spike microstructure processed under the optimal parameters contained a large amount of Cu element, while the signal of Zn element almost disappeared in the spike part, and the O element was distributed throughout the surface. This phenomenon verified the previous conjecture that at low laser power, Cu absorbs laser energy to generate corresponding oxides or hydroxides, which cover the Zn on the surface of the original ultra-fine brass wire, so the presence of Zn element was not detected in the spike structure. It can be found from Figure 6f that the signal of the Cu element detected on the surface of the spherical microstructure processed by larger laser power was significantly weakened, and the signal of the Zn and O elements were significantly enhanced, indicating that excessive laser power caused excessive etching. The oxide or hydroxide of Cu was melted and the inner layer of Zn was re-exposed. After absorbing laser energy, the oxide of Zn was formed, which leads to the enhancement of the O element signal. It can be observed from Figure 6g that the signal intensities of the Cu and Zn elements were both strong, and the signal intensity of the O element was weaker than that of the spherical microstructure. This is because at this time, the spike microstructure has not been completely melted, and a part of the original ultra-fine brass surface was exposed. The exposed part does not absorb strong enough laser energy and was not completely converted into the corresponding oxide, so that the content of the O element was lower than that of the spherical microstructure.

Figure 6d was the XRD diffraction patterns of the ultra-fine brass wire after processing under the optimal laser parameters. It can be seen that the diffraction peaks at 2*θ* = 42.02°, 48.88°, 71.86°, and 87.28° belong to the (111), (200), (220), and (311) crystal planes of CuZn-α, respectively. The diffraction peaks at 2*θ* = 41.72° and 79.82° belong to the (110) and (211) crystal planes of CuZn-β [51]. In addition to the characteristic diffraction peaks of copper–zinc alloy, there are several additional diffraction peaks. The diffraction peak at 2*θ* = 38.71° is attributed to the (111) crystal plane of CuO [52], and the diffraction peaks at 2*θ* = 43.17° and 61.84° are attributed to the (200) and (220) crystal planes of Cu_2_O [53], indicating that during laser processing, copper undergoes oxidation reaction and generates corresponding oxidation products.

### 3.6. Surface Roughness Analysis

**Figure 7:**
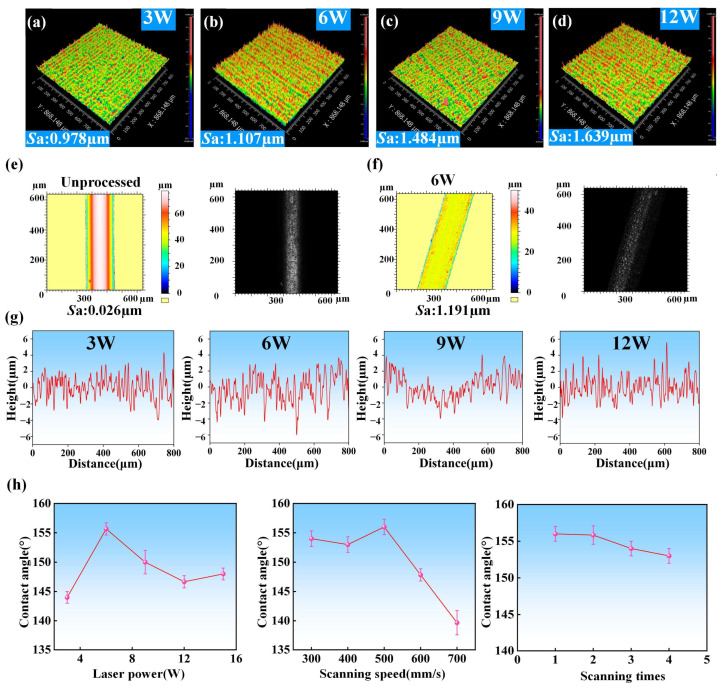
Surface roughness analysis of ultra-fine brass wire: (**a**) Zygo image of sample processed by laser power of 3 W, (**b**) Zygo image of sample processed by laser power of 6 W, (**c**) Zygo image of sample processed by laser power of 9 W, (**d**) Zygo image of sample processed by laser power of 12 W, (**e**) laser confocal microscopy images of unprocessed sample, (**f**) laser confocal microscopy images of the sample processed by laser power of 6 W, (**g**) line roughness of samples processed by different laser power (3–12 W), and (**h**) CA changing with different laser parameters.

In order to further study the influence of laser parameters on the surface roughness of the ultra-fine brass wire during processing and the subsequent influence on wettability, we set the laser scanning speed to 500 mm/s and the scanning time of 1, and use Zygo to measure the surface microstructure formed under different laser powers. Since Zygo cannot directly measure the diameter of 200 μm ultra-fine brass wire, we used brass plates with the same material composition and processed them under the same laser processing parameters instead of measuring. The measurement results were shown in Figure 7a–d. It can be clearly seen from the image that as the laser power increased, the surface roughness of the sample increased from 0.978 μm to 1.639 μm when the laser power raised from 3 W to 12 W, and from the Zygo image, the spike structure mentioned above could also be seen on the surface of the sample, that is, the red spike part in the figure. At the low laser power of 3 W, due to the weak laser energy, the processing degree of the material surface was limited, so the surface roughness was low and the number of micro/nano-structure was small. At the laser power of 6 W, the surface of the sample was covered with dense and uniform spike micropillars, and the distance between each two spike micropillars was very small, so it can maintain the appropriate roughness when the surface micro-/nano-structure was the most. As the laser power further increased, the excessive laser energy caused the surface roughness to be too large, and the spike microstructure gradually disappeared. At the same time, there appeared some large machining pits on the surface of the sample. For further analysis, we also used laser confocal microscopy to measure the surface microscopic images of unprocessed ultra-fine brass wires and processed ultra-fine brass wires under optimal laser processing parameters, as shown in Figure 7e,f. The unprocessed ultra-fine brass wire has a smooth surface and no micro/nano-structure, and its roughness was only 0.026 μm. The surface roughness of the sample obtained under the optimal laser processing parameters increased to 1.191 μm. At the same time, more micro/nano-structures could be seen in the microscopic image of the surface, which provides a good condition for constructing the superhydrophobic surface. The specific data of surface roughness under different laser processing parameters were shown in Tables S2–S4 of Supplementary Materials.

We also used Zygo to further analyze the surface morphology of the sample, including the number, height, distribution, and laser etching depth of the microstructures, as shown in Figure 7g. When the laser power was 3 W, the number of microstructures formed on the surface of the material was the least, and its height, position distribution, and laser etching depth were very uneven. Obviously, such a structure cannot meet the requirements of superhydrophobic surfaces. When the laser power was 6 W, the number of micro/nano-structures on the surface of the sample increased significantly, the height of each spike micropillar was not much different, and the uniformity of its position distribution was greatly improved. The laser processing depth was also not much different, so the sample under this parameter has excellent superhydrophobic characteristics. When the laser power increased to 9 W, although the number of microstructures did not change much, the height of the surface micropillars varied greatly. There were both large micropillars with the height of more than 4 μm and small micropillars with the height of about 1 μm. The uniformity of the position distribution became worse, and the laser etching depth also increased significantly compared with the former, so the superhydrophobic performance decreased. This also explained the phenomenon that increasing the laser power mentioned above will change the shape of the micro/nano-structure on the surface of the sample from a spike to a mastoid. When the laser power was 12 W, the number of microstructures did not change significantly, the height of the micropillars became very small, and the uniformity of the distribution position did not change much compared with before. Interestingly, the laser etching depth was very large. This is because the originally formed spike and mastoid microstructures were further etched, retaining only a small part of the bottom. In this case, the surface did not possess superhydrophobic characteristics. Through the above various characterizations, the mechanism behind the explanation was explored and explained. Finally, the law of the water contact angle changing with laser parameters was obtained as shown in Figure 7h. The specific data of the water contact angle of the sample surface under different laser processing parameters were shown in Tables S5–S7 of Supplementary Materials. When the laser power increased, the water contact angle increased first and then decreased, reaching its maximum value at 6 W. When the scanning speed increased, the water contact angle also showed a trend of increasing first and then decreased. When the scanning speed reached 500 mm/s, the water contact angle was the largest. As the scanning times increased, the water contact angle gradually decreased.

### 3.7. Application

**Figure 8:**
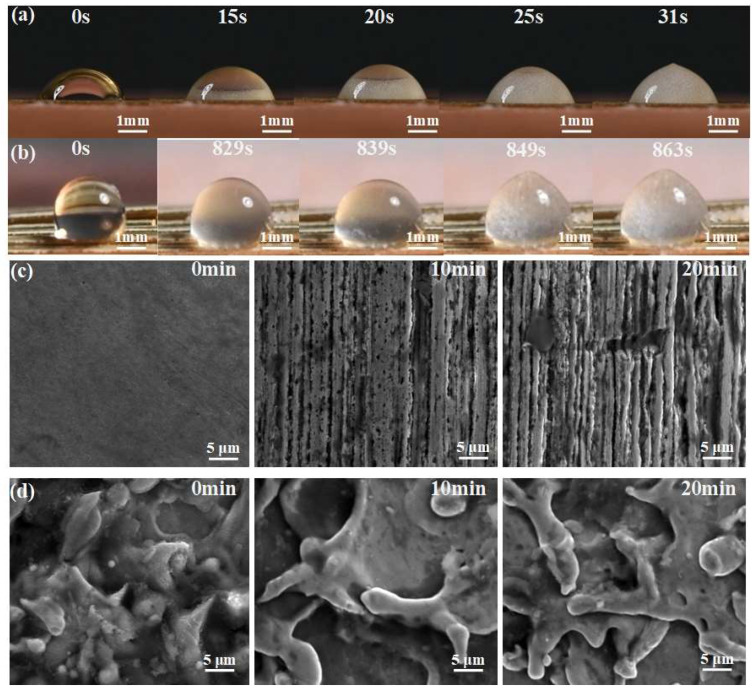
Application of superhydrophobic ultra-fine brass wire: (**a**) anti-icing test of untreated ultra-fine brass wire, (**b**) anti-icing test of superhydrophobic ultra-fine brass wire, (**c**) anti-corrosion test of untreated ultra-fine brass wire, and (**d**) anti-corrosion test of superhydrophobic ultra-fine brass wire.

In order to demonstrate the application of superhydrophobic ultra-fine brass wire in anti-icing and anti-corrosion, we designed corresponding experiments. Firstly, 5 μL droplets were dropped on the superhydrophobic ultra-fine brass wire and the untreated ultra-fine brass wire, respectively, and then placed on a water-cooled table (iCooler-4006, Shenzhen labtemp Instrument Technology Co., Ltd., Shenzhen, China). The anti-icing test was carried out under the conditions of ambient temperature 25 °C, humidity 80%, and a sample surface temperature of −16 °C. As shown in Figure 8a, on the untreated ultra-fine brass wire, the water droplets began to freeze at 15 s and completely freeze at 31 s. The whole freezing process lasted for 16 s. As shown in Figure 8b, the water droplets on the superhydrophobic ultra-fine brass wire showed signs of freezing at 829 s and completely frozen at 863 s. This can fully prove that the superhydrophobic ultra-fine brass wire has an improvement in anti-icing compared with the untreated ultra-fine brass wire.

We used 0.1 mol/L HCl solution to test the corrosion resistance of untreated ultra-fine brass wire and superhydrophobic ultra-fine brass wire. The results are shown in Figure 8c,d. It can be seen that after soaking for 10 min, the surface of untreated ultra-fine brass wire appears as a striped corrosion gully. After soaking for 20 min, the gully becomes wider and pitting pits appear. However, the surface microstructure of the superhydrophobic sample did not change significantly after immersion. It can be proved that the superhydrophobic ultra-fine brass wire has excellent corrosion resistance.

## 4. Conclusions

In this work, we proposed a simple and efficient method for processing superhydrophobic ultra-fine metal wires. Firstly, we analyzed the mechanism of laser processing of curved surfaces, explained the phenomenon of uneven quality of laser processing on curved surfaces, explored and optimized the appropriate processing scheme, and designed a special laser processing fixture for ultra-fine brass wires. It is proposed that the surface of ultra-fine brass wire could be fabricated by nanosecond laser processing, and the spikes of the micro/nano-structure could be constructed on the surface. After low surface energy modification, excellent superhydrophobic properties could be obtained. The effects of laser power, scanning speed, and scanning times on the surface microstructure and wettability of ultra-fine brass wire were studied. When the laser power was 6 W, the scanning speed was 500 mm/s, and the scanning time was 1, the superhydrophobic performance of ultra-fine brass wire was optimal, and the water contact angle was 156°. At the same time, by adjusting the laser parameters, the micro/nano-structure on the surface of the ultra-fine brass wire could be changed from spikes to mastoid and then to spherical. According to XRD and EDS, the surface of brass wire after nanosecond laser processing was mainly CuO and Cu_2_O. The influence of laser parameters on the surface roughness of ultra-fine brass wire during processing was further studied by Zygo and CLSM. By adjusting the laser parameters, the surface roughness could be increased from 0.978 μm at low power (3 W) to 1.639 μm at high power (12 W). When 6 W laser power was used for processing, the number of microstructure on the surface of ultra-fine brass wire was large, the height was moderate, and the distribution position was optimal, which laid a foundation for the subsequent realization of superhydrophobic surface. We carried out an anti-icing test on the prepared superhydrophobic ultra-fine brass wire. The test results showed that the superhydrophobic ultra-fine brass wire completely froze at −16 °C for 863 s (31 s for ordinary ultra-fine brass wire), which proved the excellent anti-icing performance of superhydrophobic ultra-fine brass wire. We also carried out the corrosion resistance test. The superhydrophobic ultra-fine brass wire did not show obvious corrosion after soaking in 0.1 mol/L HCl for 20 min, while the ordinary ultra-fine brass wire showed gully-like corrosion pits after 10 min, which proved its advantages in corrosion resistance. The final superhydrophobic ultra-fine brass wire has broad application prospects and is expected to be applied in the fields of bionic underwater robots, MEMS chip processing, and biomedicine.

## Data Availability

Data are contained within the article and Supplementary Materials.

## Supplementary Materials

The following supporting information can be downloaded at https://www.mdpi.com/article/10.3390/ma18071420/s1. Figure S1: Amplification diagram of *α′* changing with *x*.; Table S1: Surface roughness data measured at different position; Table S2: Surface roughness data measured at different laser powers; Table S3: Surface roughness data measured at different scanning speeds; Table S4: Surface roughness data measured at different scaning times; Table S5: The contact angles data measured at different laser powers; Table S6: The contact angles data measured at different scanning speeds; Table S7: The contact angles data measured at different scaning times.
