# Supplementary material for: Fabrication of Superhydrophobic Ultra-Fine Brass Wire by Laser Processing
Source: Materials (Basel). 2025 Mar 23;18(7):1420. doi: 10.3390/ma18071420 (PMC11989576; doi:10.3390/ma18071420)
Supplement: Supplementary file 1 [file materials-18-01420-s001.zip › materials-3511061-supplementary.pdf]

Tables

Table S1. Surface roughness data measured at different position.

Position	*S*a / µm	Average value / µm	Standard Deviation
A	1.157<br>1.192<br>1.187<br>1.176<br>1.185	1.179	0.0123
B	1.168<br>1.159<br>1.188<br>1.193<br>1.181	1.178	0.0126
C	1.187<br>1.178<br>1.190<br>1.166<br>1.153	1.175	0.0137
D	1.195<br>1.188<br>1.163<br>1.177<br>1.174	1.179	0.0112

Table S2. Surface roughness data measured at different laser powers.

Power / W	*S*a / µm	Average value / µm	Standard Deviation
3	0.941<br>0.960<br>0.979<br>0.996<br>1.016	0.978	0.0263
6	1.131<br>1.088<br>1.104<br>1.068<br>1.145	1.107	0.0280
9	1.464<br>1.431	1.484	0.0337

	1.515<br>1.494<br>1.522		
12	1.559<br>1.611<br>1.638<br>1.668<br>1.718	1.639	0.0534
15	2.141<br>1.989<br>2.112<br>2.098<br>1.994	2.067	0.0630

Table S3. Surface roughness data measured at different scanning speeds.

Scanning speed / mms^{-1}	*S*a / µm	Average value / µm	Standard Deviation
300	2.365<br>2.129<br>2.146<br>2.151<br>2.144	2.187	0.0998
400	1.884<br>1.844<br>1.887<br>1.944<br>1.981	1.908	0.0485
500	1.131<br>1.088<br>1.104<br>1.068<br>1.145	1.107	0.0280
600	0.995<br>0.979<br>0.972<br>0.950<br>0.958	0.971	0.0158
700	0.717<br>0.730<br>0.772<br>0.663<br>0.714	0.7192	0.0390

Table S4. Surface roughness data measured at different scaning times.

Scanning times	*S*a / μm	Average value / μm	Standard Deviation
1	1.131<br>1.088<br>1.104<br>1.068<br>1.145	1.107	0.0280
2	1.674<br>1.781<br>1.698<br>1.822<br>1.754	1.746	0.0540
3	1.942<br>1.869<br>1.937<br>1.901<br>1.895	1.909	0.0276
4	2.101<br>2.084<br>2.249<br>2.056<br>2.249	2.148	0.0838
5	2.533<br>2.612<br>2.664<br>2.417<br>2.573	2.560	0.0835

Table S5. The contact angles data measured at different laser powers.

Power / W	CA / °	Average value / °	Standard Deviation
3	143<br>144<br>142<br>145<br>145	144	1.1662
6	156<br>156<br>155<br>157<br>155	156	0.7483
9	148<br>150<br>149<br>153<br>151	150	1.7205
12	147<br>148<br>147<br>145<br>148	147	1.0954
15	149<br>148<br>149<br>146<br>147	148	1.1662

Table S6. The contact angles data measured at different scanning speeds.

Scanning speed / mms^{-1}	CA / °	Average value / °	Standard Deviation
300	153<br>155<br>155<br>153<br>154	154	0.8944
400	153<br>154<br>155<br>152<br>151	153	1.4142
500	156<br>156<br>155<br>157<br>155	156	0.7483
600	146<br>149<br>149<br>147<br>148	148	1.1662
700	138<br>144<br>142<br>136<br>141	140	2.8566

Table S7. The contact angles data measured at different scanning times.

Scanning times	CA / °	Average value / °	Standard Deviation
	156		
	156		
1	155	156	0.7483
	157		
	155		
	153		
	155		
2	157	156	1.4967
	157		
	156		
	153		
	155		
3	156	154	1.4697
	153		
	152		
	153		
	152		
4	154	153	0.6325
	153		
	153		

Figures

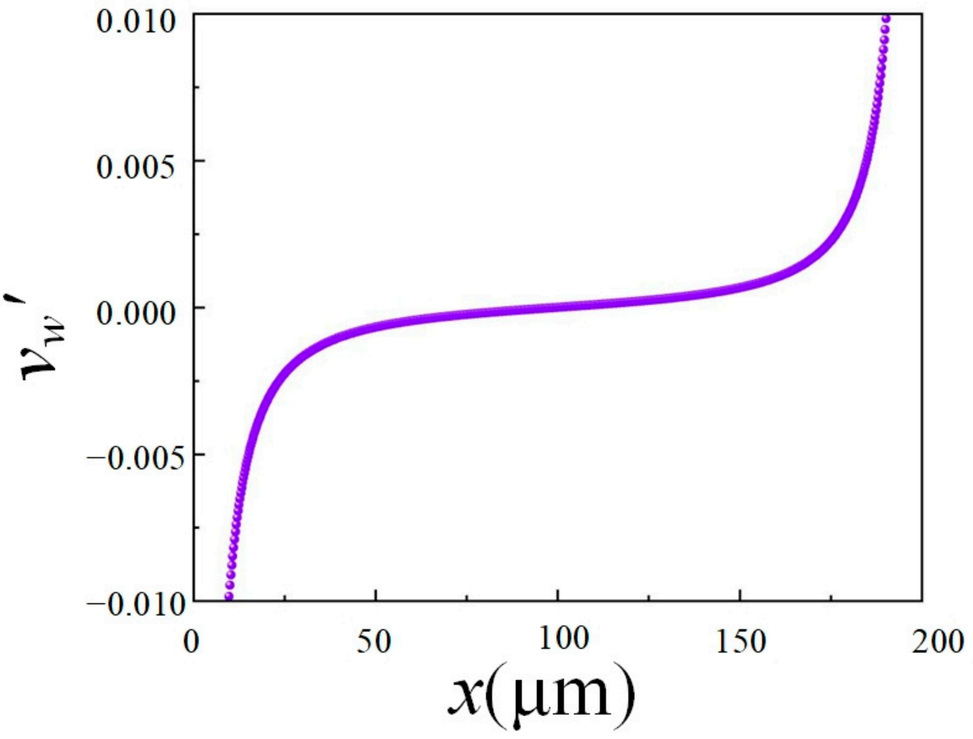

Figure S1. Amplification diagram of α' changing with x.
